# Adjuvant EGFR-TKI therapy in resected EGFR-mutation positive non-small cell lung cancer: A real-world study

**DOI:** 10.3389/fonc.2023.1132854

**Published:** 2023-03-13

**Authors:** Jun-Feng Liu, Xu-Sheng Sun, Jin-Huan Yin, Xi-E Xu

**Affiliations:** Department of Thorathic Surgery, Fourth Hospital, Hebei Meidcal University, Shijiazhuang, China

**Keywords:** adjuvant treatment, EGFR-TKI, non-small cell lung cancer, EGFR-mutation positive, adjuvant chemotherapy

## Abstract

**Background:**

Although several clinical studies have laid the foundation for the adjuvant application of epidermal growth factor receptor tyrosine kinase inhibitors (EGFR-TKIs), some questions remain unresolved. This real-world study aimed to address questions such as the effect of adjuvant chemotherapy prior to adjuvant EGFR-TKI therapy on survival outcomes, and the duration of adjuvant EGFR-TKI therapy, *etc.*

**Methods:**

Between October 2005 and October 2020, 227 consecutive patients with non-small cell lung cancer (NSCLC) who underwent complete pulmonary resections were included in this retrospective study. Patients received postoperative adjuvant chemotherapy followed by EGFR-TKI or adjuvant EGFR-TKI monotherapy. The disease-free survival (DFS) and overall survival (OS) were evaluated.

**Results:**

Of the total 227 patients, 55 (24.2%) patients underwent 3-4 cycles of chemotherapy prior to receiving adjuvant EGFR-TKI therapy. The 5-year DFS rate was 67.8%, while the 5-year OS rate was 76.4%. The stages were significantly associated with both DFS (P<0.001) and OS (P<0.001), while no significant differences were observed in the DFS (P=0.093) and OS (P=0.399) between the adjuvant chemotherapy followed by EGFR-TKI and adjuvant EGFR-TKI monotherapy groups. A longer duration of EGFR-TKI therapy was associated with better DFS (P<0.001) and OS (P<0.001) benefit. Additionally, pTNM stage and duration of EGFR-TKI therapy were considered independent prognostic factors for long-term survival (All P<0.05).

**Conclusions:**

This study supports the use of EGFR-TKI as a postoperative adjuvant treatment for patients with stage II-IIIA EGFR-mutation positive NSCLC. Additionally, patients with stage I who had pathological risk factors were also suitable for receiving adjuvant EGFR-TKI therapy. Postoperative EGFR-TKI based, chemotherapy-free adjuvant regimen may be a potential therapeutic option for patients with EGFR-mutation positive NSCLC.

## Introduction

Lung cancer is the leading cause of cancer-related deaths both in China and in other countries worldwide (1). Non-small cell lung cancer (NSCLC) accounts for 85% of all lung cancer types, and adenocarcinoma is currently the most common lung cancer subtype (2). For resectable (stage I-IIIA) lung cancer, surgical resection is the mainstay of treatment; however, 40%-60% of patients relapse within 5 years after surgery, especially patients with stage IIIA NSCLC, with a median disease-free survival (DFS) of less than 1 year (3). Therefore, it is imperative to establish an appropriate adjuvant therapy modality for these patients.

A previous meta-analysis showed that platinum-containing two-drug adjuvant chemotherapy can only increase the 5-year survival rate of patients with resectable NSCLC by 5% (4), which is recommended for stage IB-IIIA NSCLC based on the NCCN guidelines (5). However, the hematological toxicity of the platinum-containing regimen usually leads to treatment delay, dose reduction, and eventually treatment discontinuation. In recent years, the discovery of epidermal growth factor receptor (EGFR) tyrosine kinase inhibitors (TKIs) has greatly improved the efficacy of patients with advanced NSCLC harboring EGFR mutation (6). To date, several studies have shown that EGFR-TKI treatment result in a higher response rate and longer progression-free survival in advanced NSCLC compared with platinum-containing two-drug regimens (7–9).

More recently, four published randomized clinical studies laid the foundation for EGFR-TKI postoperative adjuvant application (10–12). The ADJUVANT study (10) was a phase III randomized controlled trial, which included Chinese patients with stage II-IIIA EGFR-positive NSCLC. Patients in the experimental arm were administered gefitinib once a day for 2 consecutive years after surgery, while the control arm received four cycles of vinorelbine plus cisplatin regimen postoperatively. Results showed that a significantly longer DFS was observed with adjuvant gefitinib therapy compared with vinorelbine plus cisplatin in patients with completely resected stage II-IIIA (N1-N2) EGFR-mutant NSCLC. Similarly, patients with EGFR-mutant NSCLC postoperatively receiving other EGFR-TKIs (erlotinib, osimertinib or icotinib) also observed a significantly improvement in DFS compared to chemotherapy or placebo, with a better tolerability profile (11, 12). Nevertheless, several problems were encountered in the application of EGFR-TKI after surgery, including the right stage for application, application duration, and timing of chemotherapy.

Herein, we aimed to present the results of a real-world study on postoperative adjuvant EGFR-TKI therapy in patients with EGFR-mutation positive NSCLC, with a focus on evaluating the overall survival (OS) and DFS, to address these questions.

## Materials and methods

### Study design and patients

Patients with NSCLC who underwent various curative pulmonary resections from the Fourth Hospital, Hebei Medical University were included in this retrospective study between October 2005 and October 2020. The EGFR mutations status was assessed by the ADx-ARMS EGFR Five Mutations Detection Kit (Amoy Diagnostics, Xiamen, China). Eligible patients met the following criteria (1): aged ≥18 years (2); who underwent curative pulmonary resections for NSCLC (3); with postoperative histopathological diagnosis of stage I-IIIA NSCLC based on the American Joint Committee on Cancer (AJCC) criteria, 8th edition (4); with stage IA and IB cancers who had pathological risk factors such as invasion of visceral pleura, poor differentiation, spread through air spaces (STAS), nerve or vessel invasion, and micropapillary type (5); with NSCLC harboring an EGFR-sensitive mutations (exon 19 deletion or exon 21 L858R point mutation) (6); who received adjuvant EGFR-TKI treatment with or without chemotherapy prior to adjuvant EGFR-TKI therapy (7); EGFR-TKIs initiated within 4 weeks to 16 weeks after surgery (8); with adequately functioning hematological system, liver, and kidney (9); with an Eastern Cooperative Oncology Group (ECOG) performance status of 0-1; and (10) with a postoperative survival duration of >6 months were included in the study.

Patients were excluded if they had (1) underwent palliative pulmonary resections for NSCLC (2), underwent wedge pulmonary resections with a biopsy intent (3), had concomitant cancers in other organs (4), used EGFR-TKI for less than 6 months due to certain reasons, and (5) received preoperative neoadjuvant therapy.

This study was conducted in accordance with the Declaration of Helsinki (as revised in 2013) and was approved by the Ethic Committee of Fourth Hospital, Hebei Medical University. All patients provided written informed consent.

### Adjuvant use of EGFR-TKI after complete pulmonary resection

Adjuvant EGFR-TKI therapy was initiated within 4 weeks to 16 weeks after various pulmonary resections with or without chemotherapy prior to EGFR-TKI therapy. Patients were administered with oral first-generation EGFR-TKIs including erlotinib (Roche Pharmaceuticals) 150 mg once a day, gefitinib (AstraZeneca Pharmaceuticals Ltd) 250 mg once a day, or icotinib (Betta Pharmaceuticals) 125 mg thrice a day according to the availability of these drugs in the hospital at different times. Patients received the above EGFR-TKIs continued treatment until disease progression, unacceptable toxicity, or from the study at the clinician’s discretion. Patients received postoperative chemotherapy (3-4 cycles, paclitaxel+platinum [TP] or vinorelbine+platinum [NP] regimens) before adjuvant EGFR-TKI therapy or adjuvant EGFR-TKI monotherapy. For the TP regimen, paclitaxel at a dose of 75 mg/m^2^ was administered *via* intravenous drip on day 1, while cisplatin at a dose of 15 mg/m^2^ was administered *via* intravenous drip from days 1–5, which was repeated every 3–4 weeks. For the NP regimen, vinorelbine at a dose of 25 mg/m^2^ was administered intravenously on days 1–8, while cisplatin at a dose of 75 mg/m^2^ was administered intravenously on day 1, which was repeated every 21 days.

### Follow-up

All patients were followed up every 3 months, and the computed tomography (CT) scan of the chest and upper abdomen, and the biomarkers of NSCLC were routinely examined at each follow-up visit. Brain or/and spinal magnetic resonance imaging, skeleton scan, positron emission tomography-CT scan, and even needle biopsy were performed if indicated. The patients were followed up until death or December 31, 2020. Any patient who failed to respond to two consecutive follow-up reminders was defined as “lost to follow-up.” These individuals were considered to be dead at the date of the first follow-up reminder when calculating the survival outcomes. DFS was defined as the time from surgery to the time of disease recurrence. Local or nodal recurrence and metastatic disease were considered to indicate primary tumor recurrence. OS was defined as the time from surgery to the time of death from any cause, with censoring at the longest follow-up.

### Statistical analysis

SPSS 22.0 (IBM, Armonk, NY, USA) was used to perform all statistical analyses. Continuous variables were expressed as mean ± standard deviation. Categorical variables were expressed as frequency (percentage). In the univariate analysis, DFS and OS were calculated by the Kaplan–Meier method with the log-rank test applied for comparison. Multivariate analysis using the Cox proportional hazard model (forward stepwise regression) was performed for DFS and OS. P<0.05 was considered significant.

## Results

### Patients’ and clinical data

We retrospectively analyzed 227 consecutive patients who used EGFR-TKI after undergoing complete pulmonary resections for NSCLC between October 2005 and October 2020. Of the 227 patients (84 males, 143 females; mean age, 60.8 ± 8.1 years), 96 patients had stage IA and IB NSCLC, 55 patients received 3-4 cycles of chemotherapy (TP regimen, n=32; NP regimen, n=23) prior to adjuvant EGFR-TKI therapy. The demographic and clinical data are described in **Table 1**.

**Table 1:** Patient’s demographic and clinical data.

	n=227 (%)
Sex	
Male	84 (37.0)
Female	143 (63.0)
Age (mean ± SD) years	60.8 ± 8.1
Smoke history	
Yes	23 (10.1)
No	204 (89.9)
Operation	
RUL	66 (29.1)
RML	16 (7.0)
RLL	48 (21.1)
RP	1 (0.4)
LUL	59 (26.0)
LLL	34 (15.0)
LP	3 (1.3)
Pathological type	
Adenocarcinoma	224 (98.7)
Squamous cell carcinoma	3 (1.3)
pTNM stage	
IA1	5 (2.2)
IA2	34 (15.0)
IA3	33 (14.5)
IB	24 (10.6)
IIA	3 (1.3)
IIB	47 (20.7)
IIIA	73 (32.2)
IIIB	8 (3.5)
EGFR mutation	
exon 19 deletion	113 (49.8)
exon 21 L858R	114 (50.2)
Chemotherapy before EGFR-TKI	
Yes	55 (24.2)
No	172 (75.8)
EGFR-TKI	
Erlotinib	9 (4.0)
Gefitinib	12 (5.3)
Icotinib	206 (90.7)

RUL, right upper lobectomy; RML, right middle lobectomy; RLL, right lower lobectomy; RP, right pneumonectomy; LUL, left upper lobectomy; LLL, left lower lobectomy; LP, left pneumonectomy; EGFR, epidermal growth factor receptor; TKI, tyrosine kinase inhibitors; TNM, tumor node metastasis.

### Survival

In the present study, the median follow-up was 35.23 months. Of the study patients, 36 were followed for more than 5 years, while 103 were followed for more than 3 years. The 5-year DFS rate was 67.8% (median survival time [MST] not reached), while the 5-year OS rate was 76.4% (MST not reached).

**Figure 1:**
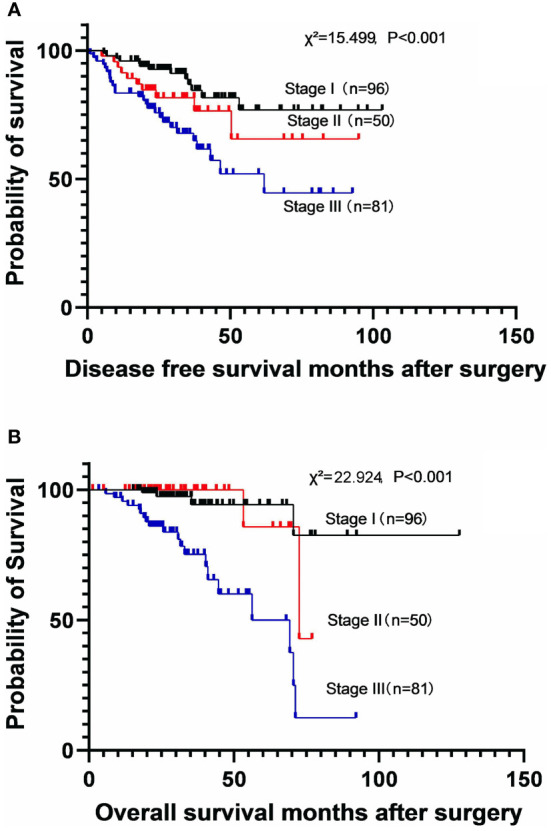
Kaplan-Meier survival curves for disease-free survival **(A)** and overall survival **(B)** according to the disease stage.

The DFS and OS were calculated according to the disease stage. The stages were significantly associated with both DFS (χ^2^ = 15.499, P<0.001) and OS (χ^2^ = 22.924, P<0.001) (**Figure 1**). The 5-year DFS rates were 74.0% (MST not reached) in stage I subgroup, 61.2% (MST not reached) in stage II subgroup, and 41.4% (MST: 43.1 months [95% CI: 32.7-53.6]) in stage III subgroup. The 5-year OS rates were 94.4% (MST not reached) in stage I subgroup, 85.7% (MST: 72.5 months [95% CI: 45.3–99.8]) in stage II subgroup, and 59.2% (MST: 69.3 months [95% CI: 52.0–86.7]) in stage III subgroup.

**Figure 2:**
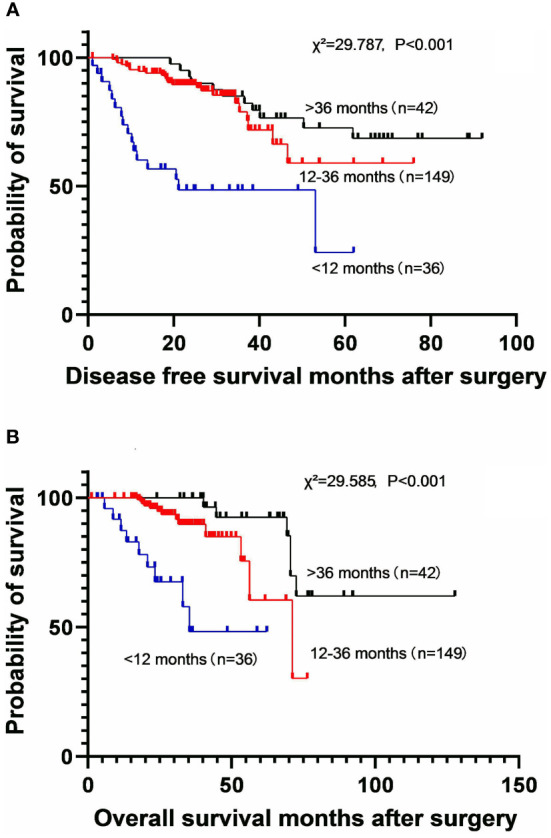
Kaplan-Meier survival curves for disease-free survival **(A)** and overall survival **(B)** according to the different durations of EGFR-TKI application.

To investigate the effect of the duration of EGFR-TKI therapy on long-term survival, the patients were divided into three subgroups, including less than 12 months subgroup, 12-36 months subgroup, and more than 36 months subgroup. A longer duration of EGFR-TKI therapy was associated with better DFS (χ^2^ = 29.787, P<0.001) and OS (χ^2^ = 29.585, P<0.001) benefit (**Figure 2**). The 5-year DFS rates were 24.3% (MST: 21.1 months [95% CI: 4.1–43.5]) in patients who used EGFR-TKIs for less than 12 months, 59.0% (MST not reached) in those who used EGFR-TKIs for 12-36 months, and 72.6% (MST not reached) in those who used EGFR-TKIs for more than 36 months. The 5-year OS rates were 48.3% (MST: 35.4 months [95% CI not estimable]) in patients who used EGFR-TKIs for less than 12 months, 60.4% (MST: 71.2 months [95% CI: 49.2-93.3]) in those who used EGFR-TKIs for 12-36 months, and 92.4% (MST not reached) in those who used EGFR-TKIs for more than 36 months.

**Figure 3:**
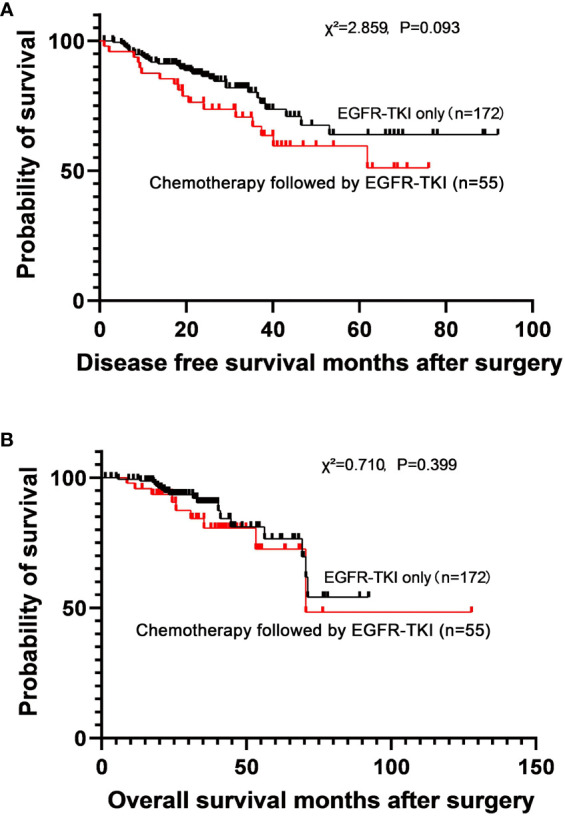
Survival curves for disease-free survival **(A)** and overall survival **(B)** in patients who used adjuvant EGFR-TKI only and adjuvant chemotherapy followed by EGFR-TKI.

Adjuvant chemotherapy was administered before adjuvant EGFR-TKI therapy in 55 patients, and 172 patients received adjuvant EGFR-TKI monotherapy. No significant differences were observed in the DFS (χ^2^ = 2.859, P=0.093) and OS (χ^2^ = 0.710, P=0.399) between the two groups (**Figure 3** *).* The 5-year DFS rates were 64.0% (MST not reached) in the adjuvant EGFR-TKI monotherapy subgroup and 59.6% (MST not reached) in the adjuvant chemotherapy followed by EGFR-TKI subgroup. The 5-year OS rates were 76.5% (MST not reached) in the adjuvant EGFR-TKI monotherapy subgroup and 72.6% (MST not reached) in the adjuvant chemotherapy followed by EGFR-TKI subgroup.

### Univariate and multivariate analysis of DFS and OS

In the univariate analysis of DFS, age (≤60 *vs.* >60 years), smoking, pathological type (adenocarcinoma vs. squamous cell carcinoma), EGFR mutations status (exon 19 deletion *vs.* exon 21 L858R), different durations of EGFR-TKI therapy, and chemotherapy prior to the adjuvant EGFR-TKI therapy were not associated with DFS. Sex, disease stage, and duration of EGFR-TKI therapy (<1 year *vs.* 1-3 years vs. >3 years) were significantly associated with DFS. Sex, disease stage, and duration of EGFR-TKI therapy (<1 year *vs.* 1-3 years *vs.* >3 years) were included in the multivariate analysis. Pathological TNM stage and duration of EGFR-TKI therapy were independent prognostic factors for DFS (**Table 2**).

**Table 2:** Results of univariate and multivariate analyses of disease-free survival and overall survival.

	Disease-free survival				Overall survival			
	Univariate analysis		Multivariate analysis		Univariate analysis		Multivariate analysis	
	HR (95% CI)	P	HR (95% CI)	P	HR (95% CI)	P	HR (95% CI)	P
Sex: male vs. female	0.441 (0.255-0.764)	0.015	0.573 (0.321-1.021)	0.101	0.470 (0.220-1.003)	0.045	0.507 (0.221-1.165)	0.110
Age: <60 vs. ≥60 years	1.005 (0.580-1.742)	0.985			1.478 (0.695-3.142)	0.210		
Smoke history: yes vs. no	1.071 (0.421-2.727)	0.886			0.938 (0.216-4.078)	0.932		
Pathologic type: adenocarcinoma vs. squamous	0.049 (0.000-3612.560)	0.472			0.048 (000-796629.128)	0.592		
pTNM stage: I vs. II vs. III	1.876 (1.348-2.610)	0.000	1.915 (1.367-2.683)	0.000	3.390 (1.922-5.978)	0.000	3.315 (1.864-5.898)	0.000
EGFR mutation: exon 19 vs. exon 21	1.382 (0.942-2.027)	0.092			1.645 (0.906-2.987)	0.119		
Chemotherapy before TKI vs. TKI only	1.176 (0.966-1.431)	0.093			1.121 (0.857-1.467)	0.399		
EGFR-TKI: erlotinib vs. gefitinib vs. icotinib	0.748 (0.481-1.165)	0.178			0.680 (0.416-1.111)	0.123		
Duration of TKI use: <1 vs. 1-3 vs. >3 years	0.399 (0.254-0.627)	0.000	0.318 (0.150-0.676)	0.000	0.246 (0.132-0.458)	0.000	0.217 (0.109-0.432)	0.000

TNM, tumor node metastasis; EGFR, epidermal growth factor receptor; TKI, tyrosine kinase inhibitors.

In the univariate analysis of OS, age (≤60 *vs.* >60 years), smoking status, pathological type (adenocarcinoma vs. squamous cell carcinoma), EGFR mutations status (exon 19 deletion *vs.* exon 21 L858R), different durations of EGFR-TKI therapy, and chemotherapy prior to the adjuvant EGFR-TKI therapy were not associated with OS. However, sex, disease stage, and the duration of EGFR-TKI therapy (<1 year *vs.* 1-3 years *vs.* >3 years) were significantly associated with OS. Sex, disease stage, and duration of EGFR-TKI therapy (<1 year *vs.* 1–3 years *vs.* >3 years) were included in the multivariate analysis. Pathological TNM stage and duration of EGFR-TKI therapy were independent prognostic factors for OS (**Table 2**).

## Discussion

Four clinical studies, including ADJUVANT, EVEN, ADAURA, and EVIDENCE, examined the efficacy of postoperative adjuvant EGFR-TKI treatment in EGFR-mutant NSCLC, which provided clinicians with several useful informations, and changed the therapeutic modes of EGFR-TKI in the clinical setting to a certain extent. However, these studies had not addressed all the issues in this field, and many questions had remained hot topics for controversies such as the effect of adjuvant chemotherapy prior to adjuvant EGFR-TKI therapy on survival outcomes, and the duration of adjuvant EGFR-TKI therapy, *etc.* As a real-world study, this research may help deal with these leftover issues.

The first point of contention is whether to use adjuvant TKI alone or adjuvant chemotherapy prior to adjuvant EGFR-TKI therapy. Adjuvant chemotherapy after surgical resection would appear to be the logical approach in order to reduce disease recurrence and improve survival (13). However, results from the EVIDENCE trial showed that adjuvant icotinib significantly improves DFS in patients with EGFR-mutant stage II-IIIA NSCLC after complete tumor resection compared with adjuvant chemotherapy (47.0 vs. 22.1 months) (12). The ADAURA trial also confirmed the DFS advantage of adjuvant osimertinib over placebo in enrolled postoperative patients, the great mass of which had received prior chemotherapy (14). Notably, adding adjuvant chemotherapy before adjuvant EGFR-TKI was not beneficial among osimertinib-based patients according to cross-arm comparison of ADAURA trial by Liang et al. (15). Similarly, the addition of 3 to 4 cycles of adjuvant chemotherapy prior to adjuvant EGFR-TKI therapy did not significantly improve the DFS (5-year DFS rates: 59.6% vs. 64.0%, P=0.093) and OS (5-year OS rates: 72.6% vs. 76.5%, P=0.399) compared with adjuvant EGFR-TKI alone in the present study. Based on these findings, adjuvant EGFR-TKI appears to be superior to adjuvant chemotherapy in terms of DFS and can result in a further improvement in DFS for patients previously treated with postoperative adjuvant chemotherapy. Besides, there seems to be no additional survival benefit of adding adjuvant chemotherapy prior to adjuvant EGFR-TKI in patient with EGFR-mutation positive NSCLC. Certainly, considering the heterogeneity of patients, further screening of the precise beneficiary population for adjuvant chemotherapy and adjuvant EGFR-TKI therapy based on biomarkers (e.g., molecular residual disease [MRD] detection) may be required to enable patients to choose the optimal individualized treatment regimen while ensuring survival.

The second focus of controversy is the duration of adjuvant EGFR-TKI therapy. Previous studies had reported that the duration of adjuvant EGFR-TKI therapy had ranged from 0.5-3 years (10–12, 16). In the ADJUVANT study, the recurrence-free survival curve showed a significant downward trend after 2 years, which might be explained by the discontinuation of EGFR-TKI. Another randomized control trial conducted by Lyu et al. showed that the 2-year adjuvant icotinib therapy observed a significantly improved DFS and OS without an increase incidence of toxicity versus comparator 1-year therapy (17). In addition, the recurrence dynamics of the resected NSCLC display a multi-peak pattern, with the first peak occurring 7-9 months postoperatively irrespective of gender, the second peak occurring earlier in men (18-20 months) than in women (24-26 months), and the third peak occurring during the fourth year (18, 19). Thus, the ideal duration of medication therapy should cover these three periods of recurrence peaks. In the present study, the 5-year OS rates for patients treated with adjuvant EGFR-TKIs for less than 12 months, 12-36 months and more than 36 months were 48.3% (MST: 35.4 months [95% CI not estimable]), 60.4% (MST: 71.2 months [95% CI: 49.2-93.3]), and 92.4% (MST not reached), respectively. This finding indicated that survival advantage of patients seemed to increase with longer duration of EGFR-TKIs administration, offering more benefits in long-term outcomes. Moreover, the duration of TKI therapy was considered an independent prognostic factor by multivariate analysis in the present study. Accordingly, we also have to concern whether lifelong use of EGFR-TKIs is necessary and whether presence of patients who had been cured from surgery. Currently, Zhang et al. reported the prognostic value of MRD detection in patients with NSCLC after surgery, suggesting that patients with longitudinal undetectable MRD represent a potentially cured population and might not benefit from adjuvant therapy (20). Given the above evidence, the necessity to continue adjuvant EGFR-TKI therapy can be identified in the future studies by screening the patient population with long-term MRD detection (≥18 months).

The third focus of attention is determining the NSCLC stage that is suitable for adjuvant EGFR-TKI therapy. Due to the results of the four clinical studies mentioned above, the postoperative adjuvant EGFR-TKI therapy should be incontrovertible in patients with stage II–IIIA NSCLC. The focus of attention is whether patients with stage I EGFR-mutant NSCLC should receive adjuvant EGFR-TKI therapy. Goldstraw et al. reported a 5-year survival rate of 73% for patients with stage IB NSCLC (21), which implied that 27% of patients with stage IB will die due to locoregional recurrence or systemic spread within 5 years. Nevertheless, adjuvant chemotherapy for patients with stage I NSCLC who had risk factors such as lymphovascular or pleural invasion was still able to obtain a survival benefit (22, 23). This suggested a survival benefit for postoperative adjuvant therapy even in patients with stage I NSCLC with risk factors such as lymphovascular or pleural invasion. Furthermore, adjuvant osimertinib therapy had been shown to provide a survival benefit in patients with stage IB by the ADAURA trial. In the present study, 5-year OS rate for patients with stage I had achieved 94.4%, which was comparable with the historical data (21). Notably, all patients with stage I included in this study had pathological risk factors such as invasion of visceral pleura, poor differentiation, STAS, nerve or vessel invasion, and micropapillary type. Overall, this study supports adjuvant EGFR-TKI therapy for patients with stage I NSCLC who had pathological risk factors (invasion of visceral pleura, poor differentiation, or STAS, *etc.*).

There were several limitations to this study. Our study was a retrospective study conducted in a single-center, selection bias was inevitable due to the inherent limitations of single-center, non-randomized and retrospective design. Besides, this non-global study with data was only conducted in China, which might affect the generalizability of the results to a broader population. Therefore, more prospective studies and longer follow-up are required to validate the efficacy and safety of EGFR-TKI based, chemotherapy-free adjuvant regimen in patients with EGFR-mutation positive NSCLC.

## Conclusion

In conclusion, this study supports the use of EGFR-TKI as a postoperative adjuvant treatment for patients with EGFR-mutation positive stage II-IIIA NSCLC. Additionally, patients with stage I who had pathological risk factors were also suitable for receiving adjuvant EGFR-TKI therapy. Postoperative EGFR-TKI based, chemotherapy-free adjuvant regimen may be a potential therapeutic option for patient with EGFR-mutation positive NSCLC. However, a multi-center randomized control trial is warranted to validate these findings.

## Data availability statement

The original contributions presented in the study are included in the article/supplementary materials, further inquiries can be directed to the corresponding author/s.

## Ethics statement

The studies involving human participants were reviewed and approved by the Ethic Committee of Fourth Hospital, Hebei Medical University. The patients/participants provided their written informed consent to participate in this study.

## Author contributions

J-FL: Conception and design; X-SS: Providing materials and samples; J-HY and X-EX: Data collection; J-FL: Data analysis and interpretation; J-FL: Drafting article; J-FL: Administrative support. All authors contributed to the article and approved the submitted version.
